# Supplementary material for: Deciphering the role of cis-regulatory elements and TFAP2C in the activation of zygotic Sox2 expression in mouse preimplantation embryos
Source: Development. 2025 Jul 18;152(14):dev204626. doi: 10.1242/dev.204626 (PMC12338974; doi:10.1242/dev.204626)
Supplement: Supplementary information [file develop-152-204626-s1.pdf]

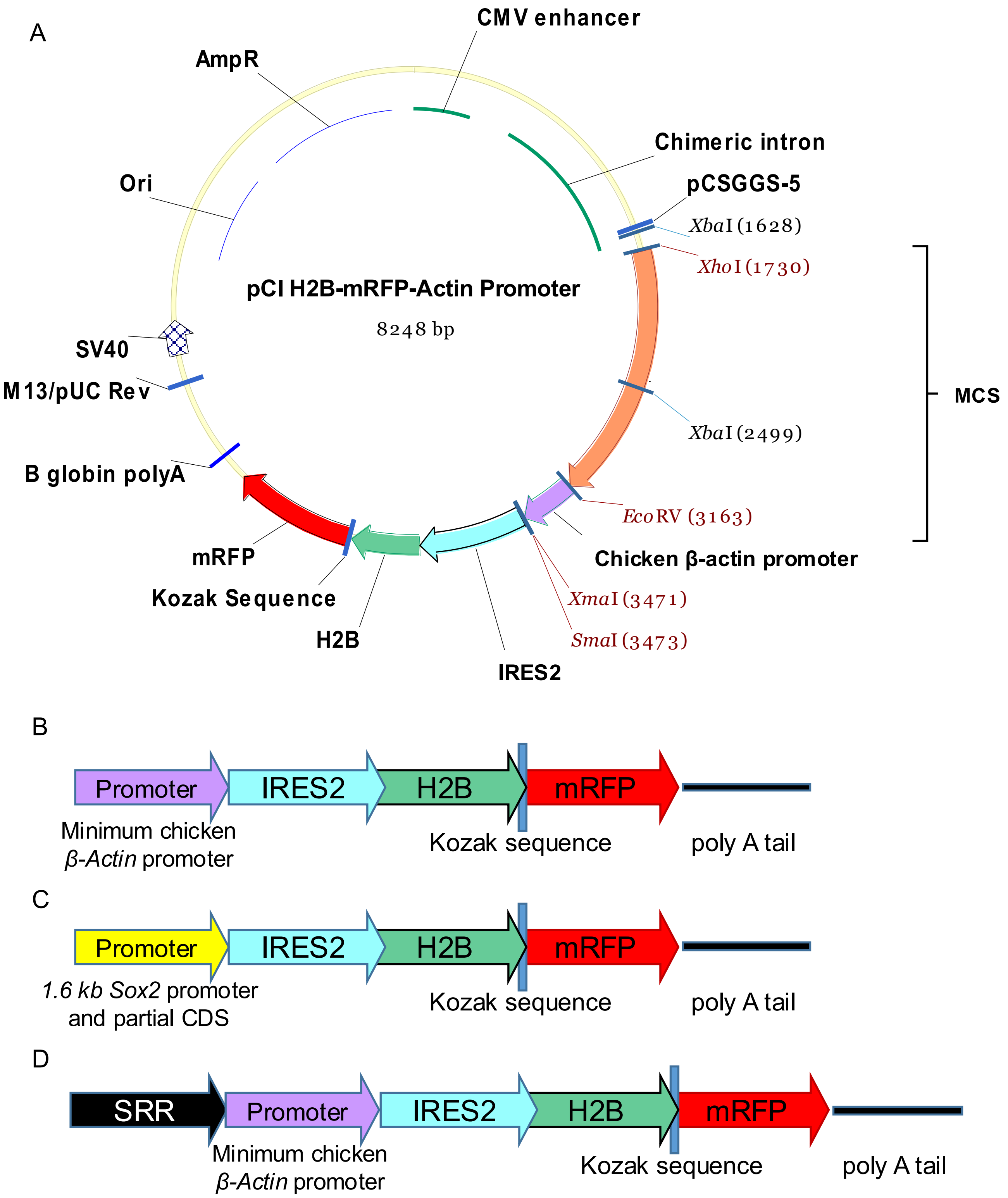

Fig. S1. pCl H2B-mRFP vector map and linear construct schematic. (A) Vector map of pCL H2B-mRFP. The minimum chicken *β-actin* promoter was introduced to enhance expression. The restriction enzymes XhoI and EcoRV were used for inserting the SRRs and promoter into the vector. (B-D) Schematic of the negative control (B), *Sox2* promoter (C) and SRR (D) reporter construct used for pronuclear injection in zygotes. H2b-mRFP expression was driven by the activity of the *Sox2* SRR and promoter cis-regulatory elements.

Development • Supplementary information

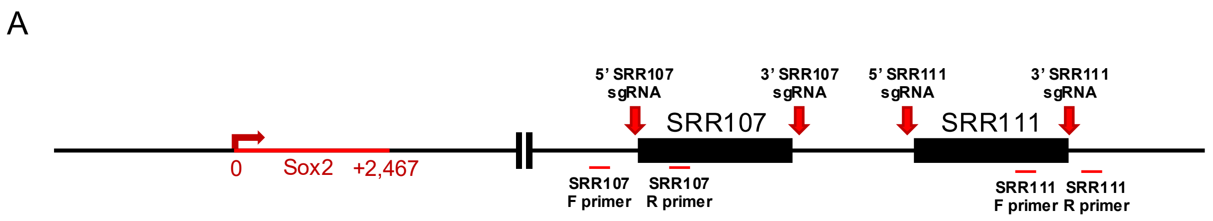

Genotyping primer	Sequence
SRR107 F	GCACAGGCTACCCTGGCAATGG
SRR107 R	TCATTTGAGTCCCTTCTTCTGG
SRR111 F	ATTCTGTGCAGGGCAATAGTGGC
SRR111 R	GCACTTGCTTGTAATCTAGCTCC

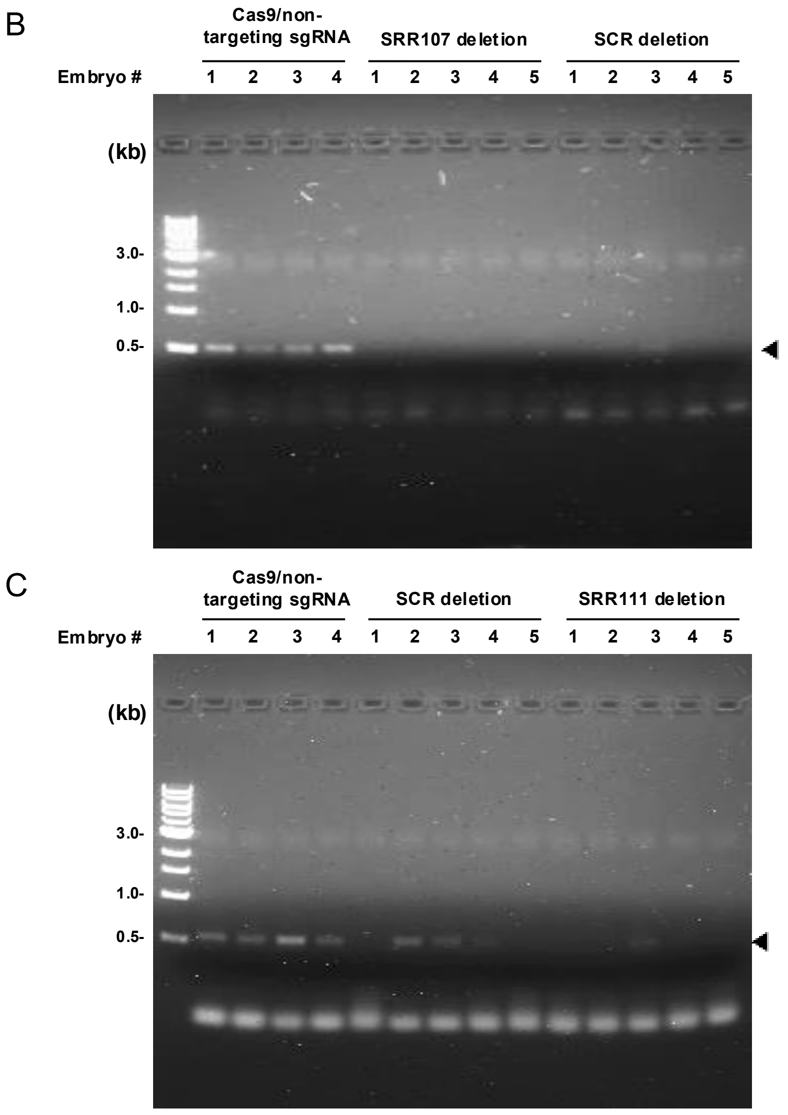

Fig. S2. CRISPR/Cas9 SRR107 and 111 genotyping results. (A) Schematic diagram of SRR107 and SRR111 (black boxes) in the mouse genome and the position of the targeting small guide RNA(sgRNA) (red arrows). SRR107 and SRR111 alone or the entire region (SCR) were targeted using the sgRNAs shown. The primer regions (red bars) for genotyping are shown in the diagram sequence. (B) Agarose gel images illustrating the PCR-based single blastocyst genotyping assay of SRR107 and SCR and (C) SCR and SRR111. The expected band size is 545 bp for SRR107 and 571 bp for SRR111.

Development • Supplementary information

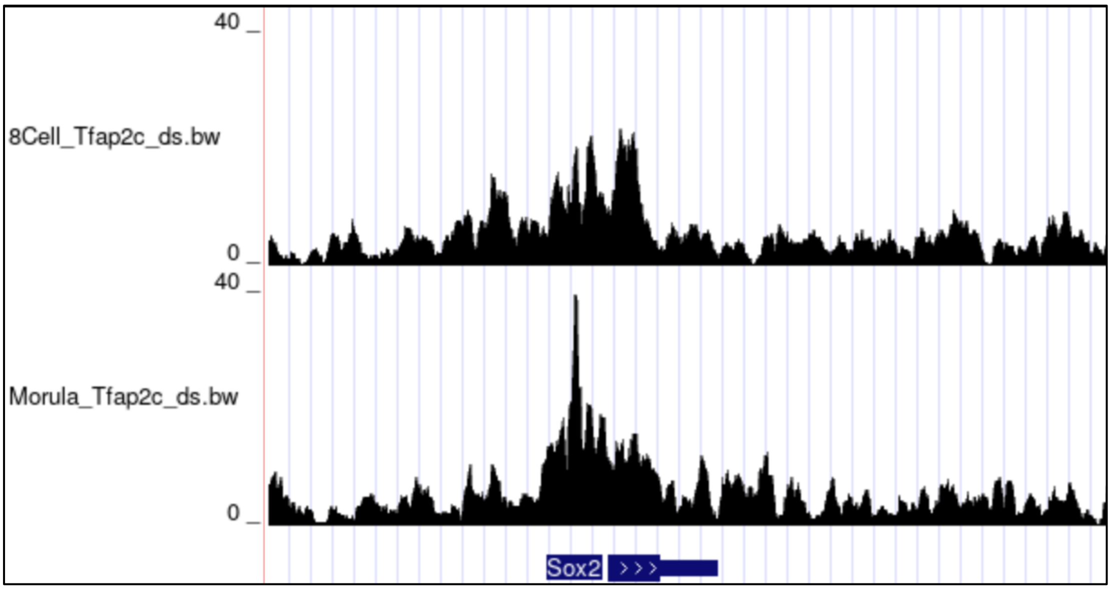

Fig. S3. CUT&RUN analysis of TFAP2C binding. The UCSC genome browser was used to examine TFAP2C binding to the *Sox2* promoter in 8-cell embryos versus morulae. TFAP2C was enriched at both the 8-cell and morula stages.

Development • Supplementary information

A

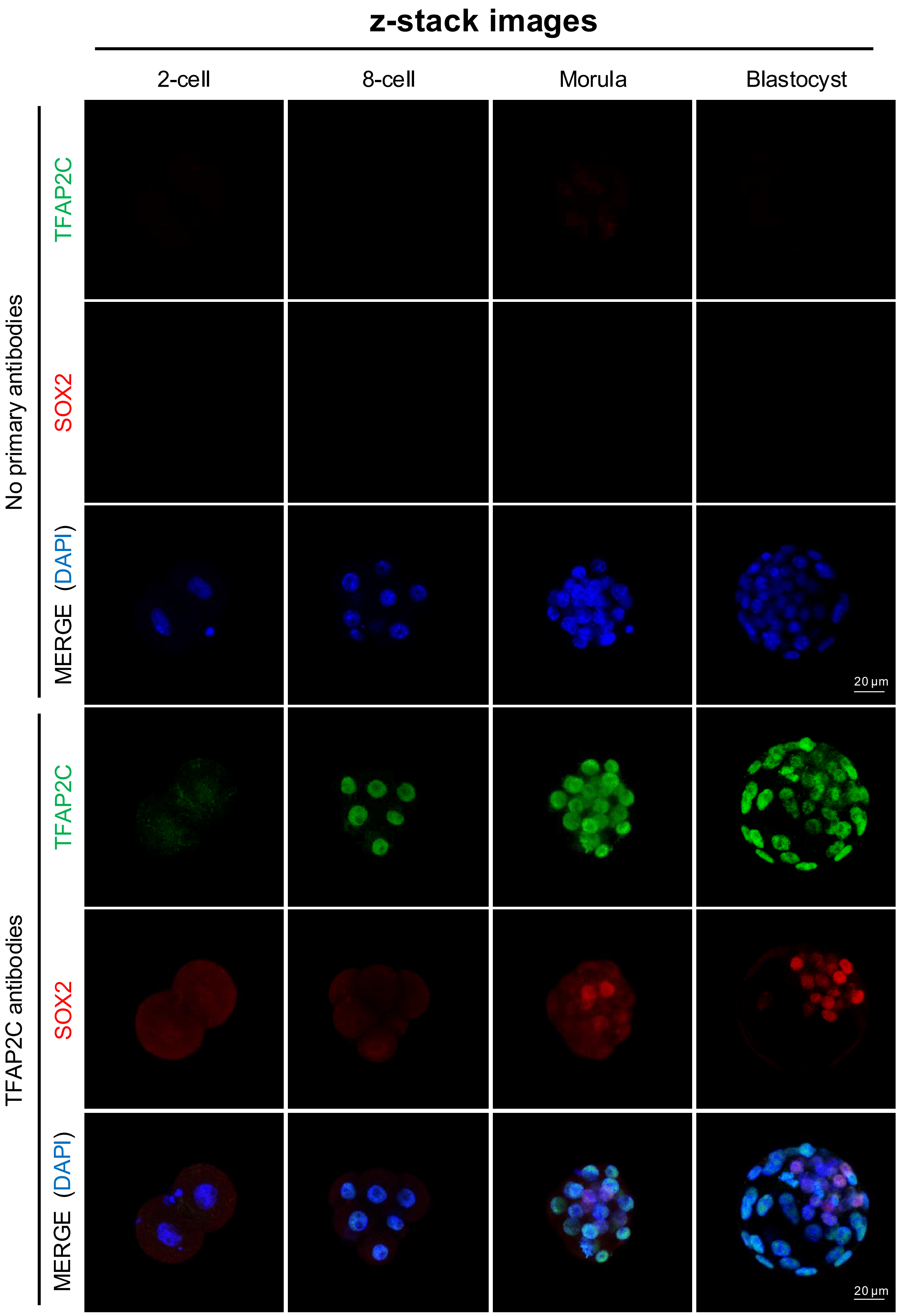
z-stack images
2-cell
8-cell
Morula
Blastocyst
No primary antibodies
TFAP2C antibodies
TFAP2C
SOX2
MERGE (DAPI)
20 μm

B

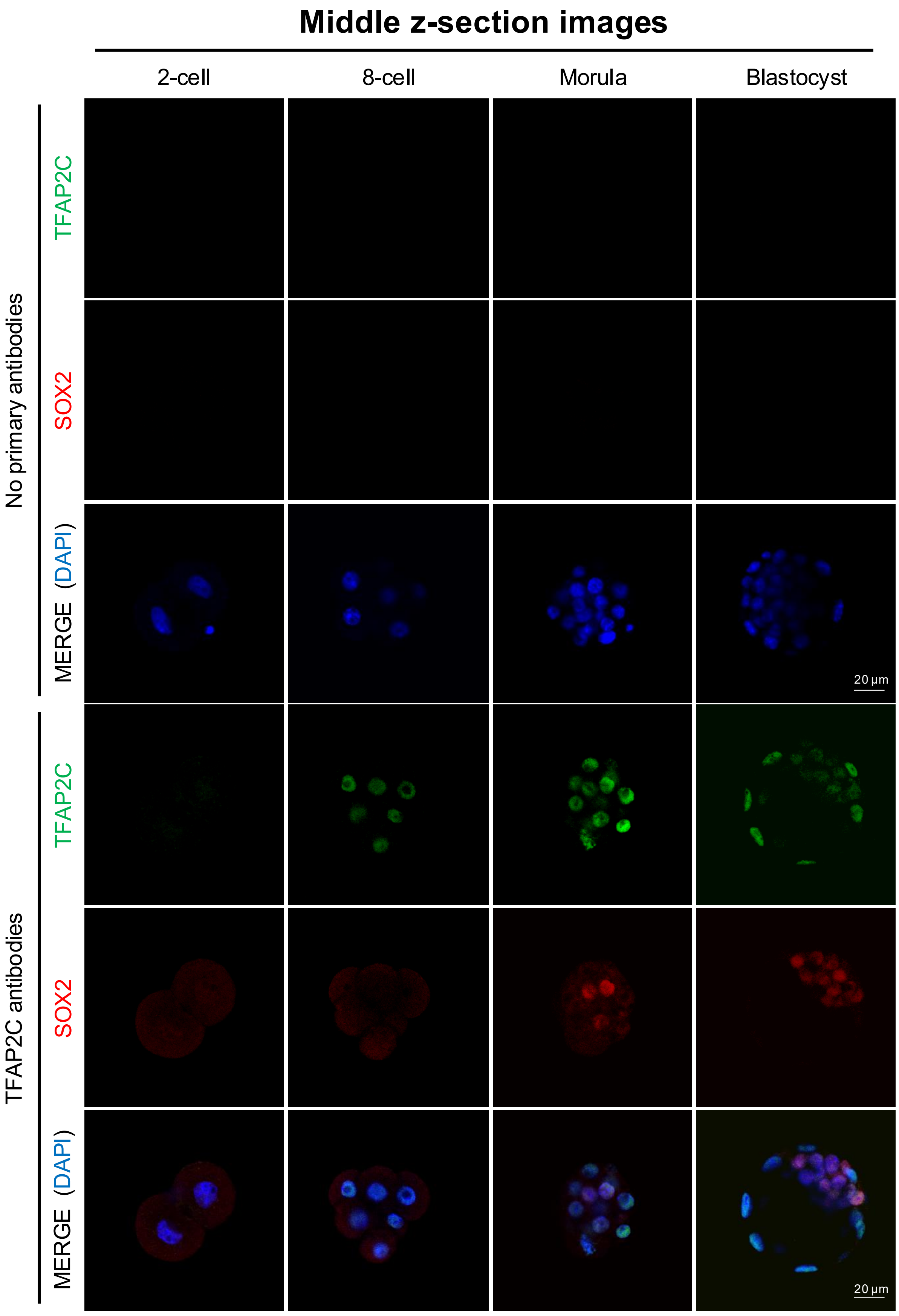

Development • Supplementary information

C

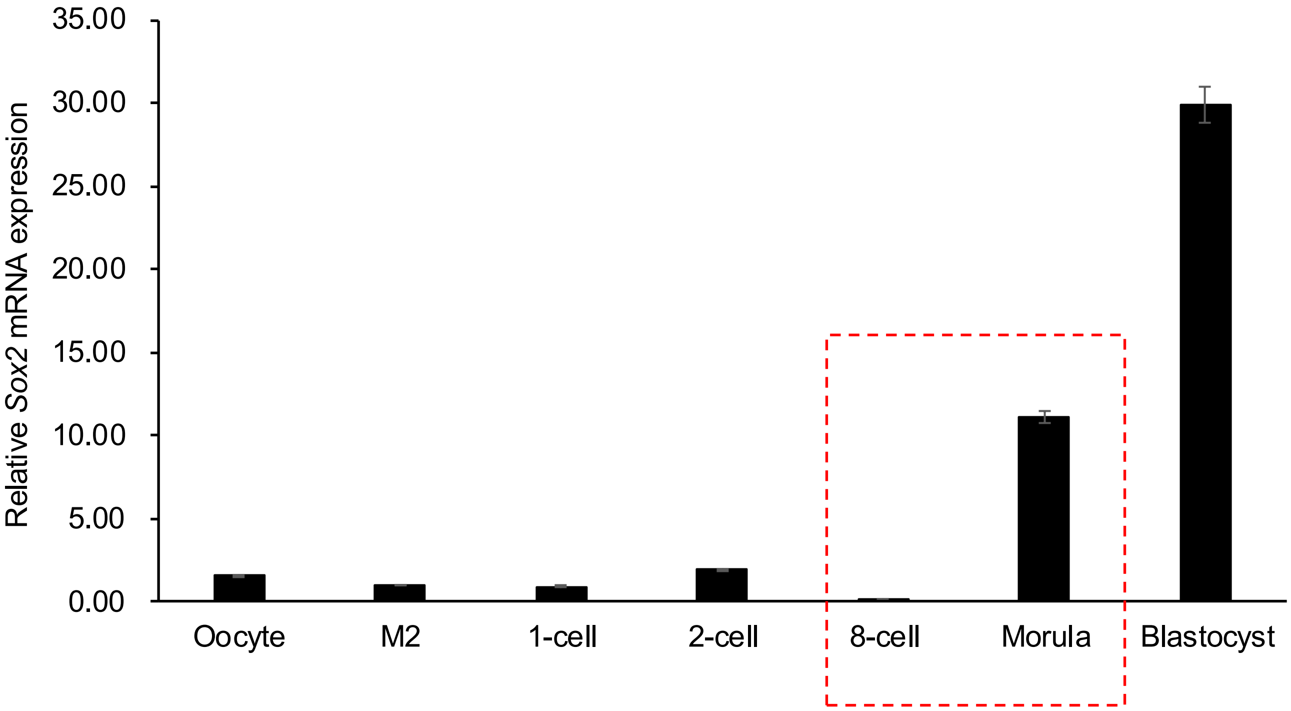

Fig. S4. Developmental expression of *Sox2* transcripts and SOX2 and TFAP2C proteins in mouse preimplantation embryos. (A) Z-stack and (B) single confocal sections of immunofluorescence images of TFAP2C and SOX2 in mouse preimplantation embryos. TFAP2C and SOX2 expression were evaluated in 2-cell (E1.5), 8-cell (2.5), morula (E3.25), and blastocyst stage (E4.5) embryos (scale bar, 20 μm). (C) Real-time PCR analysis of *Sox2* transcripts in oocytes and preimplantation embryos. Dashed box highlights the increase in zygotic *Sox2* mRNA between the 8-cell and morula stages. Exogenous *hmGFP* and embryo number were used to normalize *Sox2* expression. Values mean ± SD. A total three biological replicates were used.

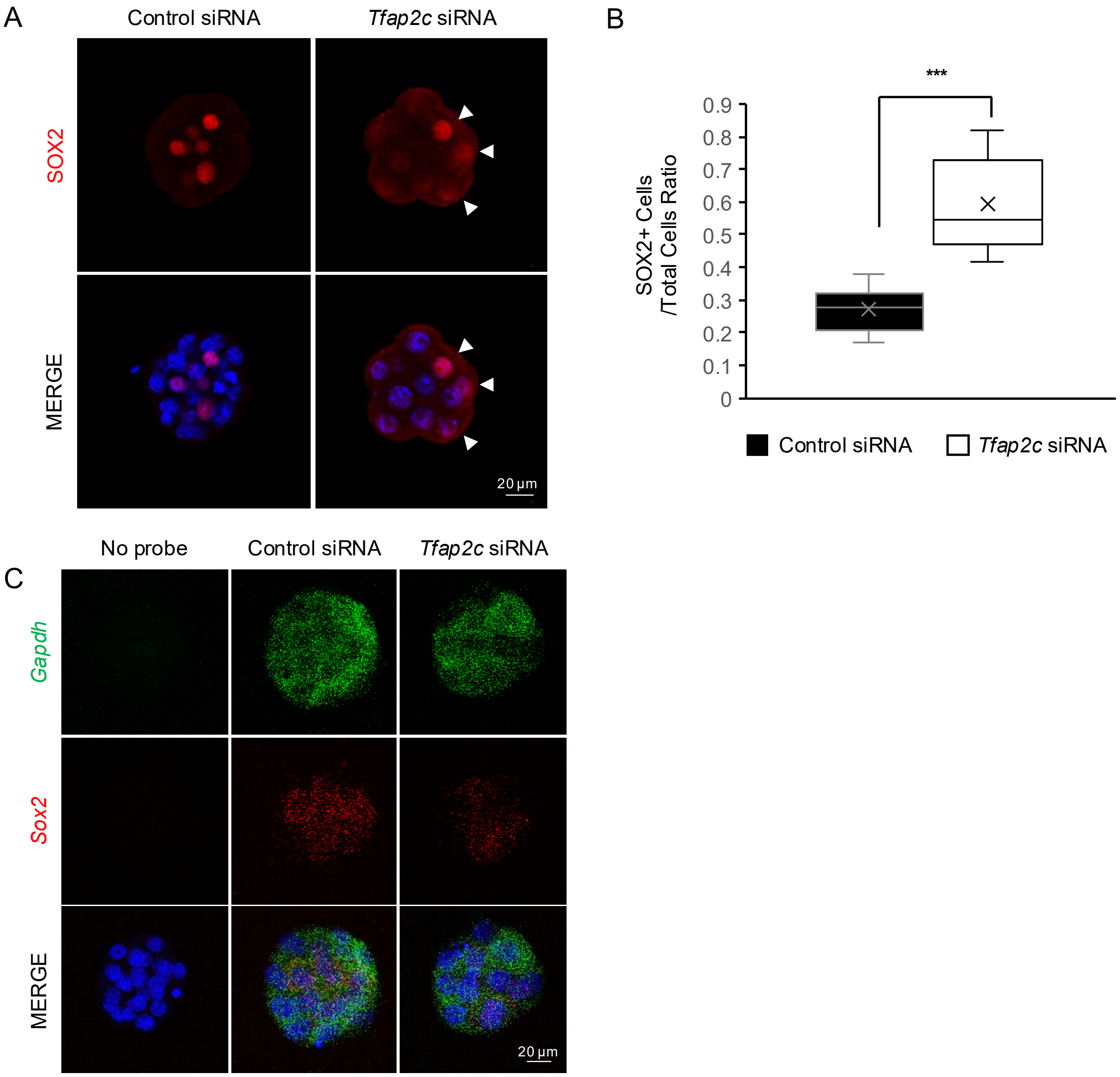

Fig. S5. Effects of *Tfap2c* knockdown on *Sox2* expression.

(A) Confocal immunofluorescence analysis of SOX2 expression and localization in control siRNA and *Tfap2c* siRNA injected embryos at the morula stage (E3.25). Ectopic SOX2 expression was observed in the apical region of embryos (white arrowheads; scale bar, 20 μm). (B) Ratio of SOX2 positive cells to total cell number in control siRNA and*Tfap2c* siRNA injected embryos. This analysis was carried out using Z-stack images from n=8 control siRNA and n=8 *Tfap2c* siRNA injected embryos. Embryos were counterstained with DAPI. Values are presented as means ± SD. *** P<0.001 (Student's t-test). (C) RNA-ISH images of *Sox2* mRNA in control (n=7) and *Tfap2c* KD embryos (n=6) at morula stage (E3.25). *Gapdh* mRNA was used as a positive control (scale bar, 20 μm).

Development • Supplementary information

A

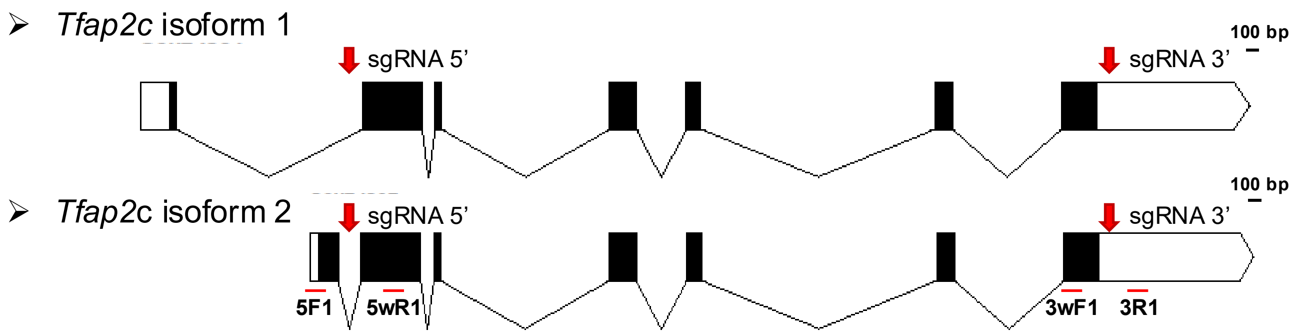

Genotyping primer	Sequence
Tfap2c 5' F1	CCGATAGGCTCACAACGAAGTCC
Tfap2c 5' R1	CTCACCTGCACCTCCTCTATGG
Tfap2c 3' F1	GACACCCAACGGGAACAACAGG
Tfap2c 3' R1	CCAGAAGGTGTGATTGACAAGG

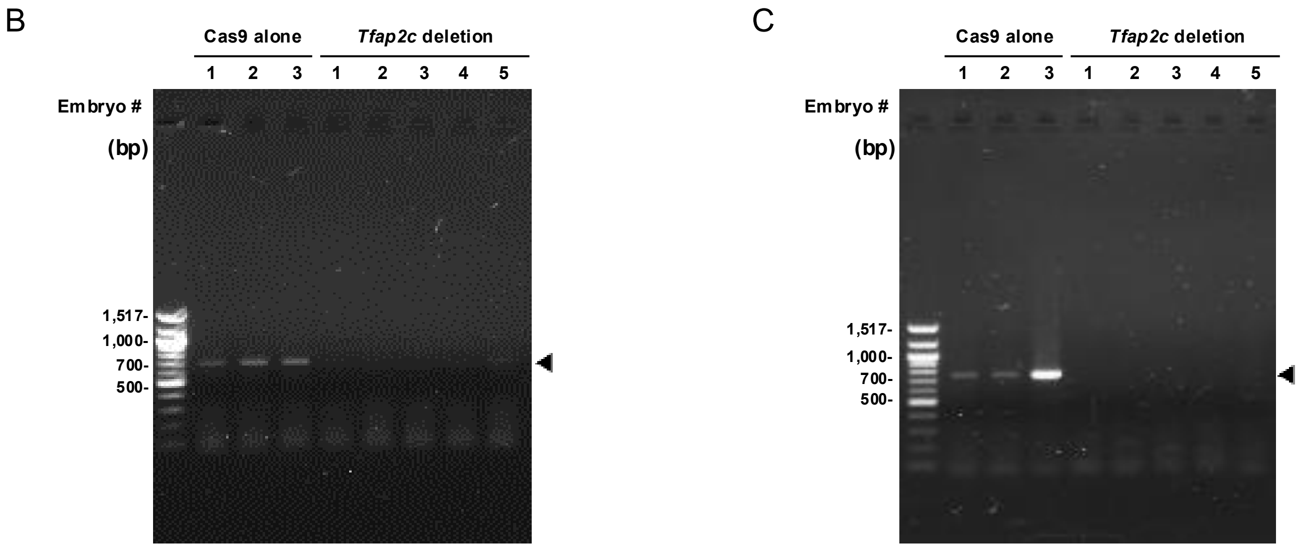

Fig. S6. *Tfap2c* CRISPR/Cas9 genotyping results.

(A) Schematic diagram of two *Tfap2c* isoforms including exons (black boxes) and single-guide RNA (sgRNA) positions (red arrows). The primer regions (red bar) for genotyping are indicated in the diagram sequence. (B) Agarose gel images illustrating the PCR-based 5’ region genotyping assay of *Tfap2c* deleted embryos at the morula stage (E3.25). The expected band size is 665 bp. (C) Agarose gel images illustrating the PCR-based 3’ region genotyping assay of *Tfap2c* deleted embryos. The expected band size is 751 bp.

Development • Supplementary information

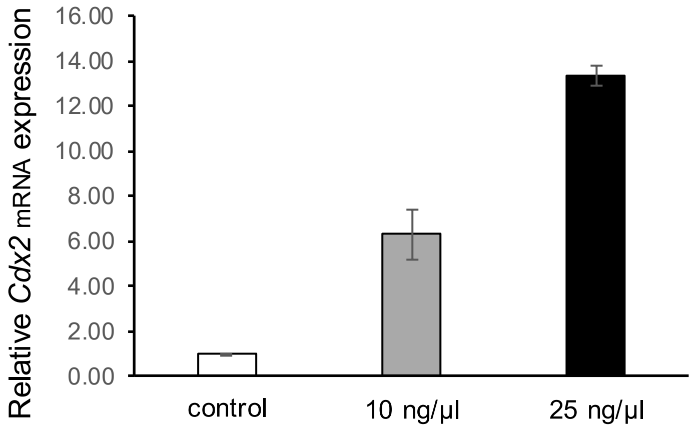

Fig. S7. Effects of *Tfap2c* cRNA on *Cdx2* expression.

Real-time PCR analysis of *Cdx2* transcripts in control versus *Tfap2c* cRNA injected embryos at the 2-cell stage (E1.5). Two biological replicates were used, with 30 pooled embryos per replicate. Values are means ± SD.

Development • Supplementary information

A

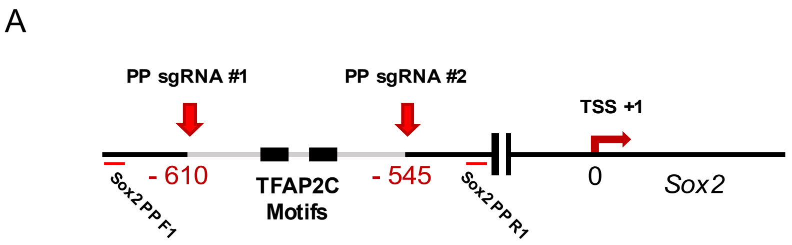

Genotyping primer	Sequence
Sox2 PP F1	TGAGGACACGTGCTGCGGTTCC
Sox2 PP R1	ACCAAGACGACAGCTCCTTTCC

B

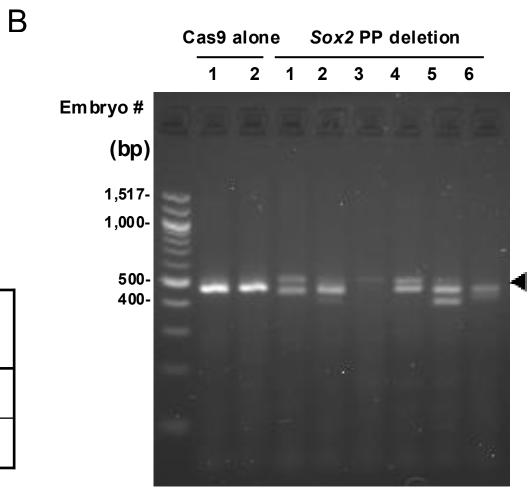

C

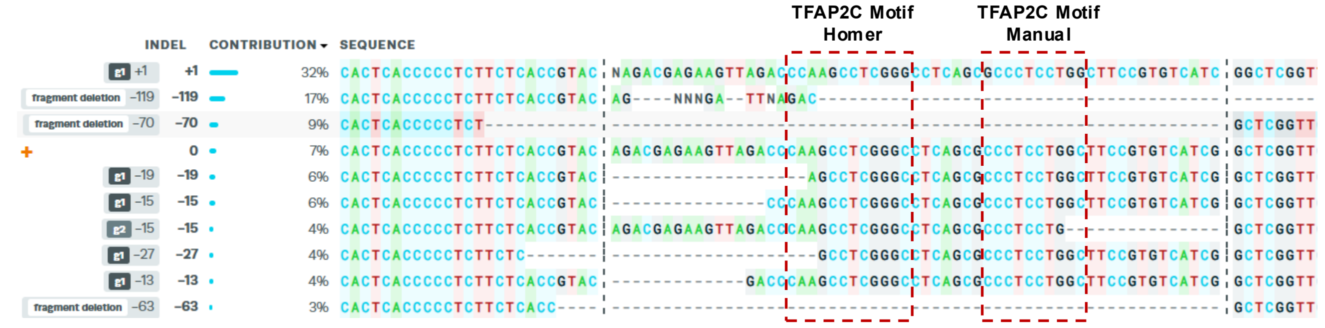

Fig. S8. ***Sox2*** **PP TFAP2C motif editing and genotyping results.**
(A) Schematic diagram of two TFPA2C binding motifs and single-guide RNA (sgRNA) positions (red arrows). The primer regions (red bar) for genotyping are indicated in the diagram sequence. (B) 3% agarose gel image illustrating the PCR-based *Sox2* PP deletion genotyping assay at the blastocyst stage (E4.5). The expected band size is 457 bp. (C) An example of a sequencing result for a *Sox2* PP edited embryo. The TFPA2C binding motifs are outlined in the red squares.

Development • Supplementary information

Table S1. *In silico* analysis of TFAP2C binding motifs in *Sox2* cis-regulatory elements

Region	Location	Length	Distance from TSS	Expected TFAP2C binding region	Sequence
SRR1	Ch.3 34,700,234~34,700,684	451	-3937~-3487	None	
Sox2 1.6kb Promoter/ partial CDS	Ch.3 34,703,499~34,705,060	1562	-1066~+496	-1,015~-1,006	GCCAGGAGGG
				-998~-990	GCCCGAGGC
				-268~-260	GCCCGAGGC
				-101~-93	GCCCGCGGC
				-33~-25	GCCGCCGGC
				-30~-21	GCCGGCGGGC
				+30~+39	GCCGCCGGGC
				+286~+295	GCCAAGCGGC
				+403~+411	GCCCCCGGC
SRR2	Ch.3 34,707,864~34,708,245	382	+3,641~+4,023	+3,697~+3,706	GCCTCTAGGC
SRR107	Ch.3 34,811,798~34,813,334	1,537	+107,654~+109,191	+107,666~107,674	GCCAAAGGC
				+107,936~107,945	GCCGGGAGGG
SRR111	Ch.3 34,815,172~34,816,684	1,513	+111,028~+112,541	+111,602~111,610	GCCAAAGGG
				+113,038~113,046	GCCCTGGGC

Table S2. Control and *Tfap2c* siRNA sequences

Target	Sequence
Tfap2c SMART pool siRNAs	AAGCUGAGUUCCCUAGUAA, GCACGGGACUUCGCCUAUG, AGCGGUGGCUGACUAUUUA, CCGCAGUGCAGAAUUAUAU
Control non-targeting siRNAs	UAAGGCUAUGAAGAGAUAC, AUGUAUUGGCCUGUAUUAG, AUGAACGUGAAUUGCUCAA, UGGUUUACAUGUCGACUAA

Development • Supplementary information

Table S3. Custom-designed sgRNAs for CRISPR deletion

Target region	Sequence
5' *Tfap2c* exon2	GCUCGAGUCGUGGCGAUCCU
3' *Tfap2c* exon7	AAUCCGUUCCCUCUCUUGAA
5’ SRR107	ACAAAAACAUGUACGUUGGG
3’ SRR107	CCGUCCCAUCCACUGUAUUA
5’ SRR111	GACAUAUCAAUGACUAGGCG
3’ SRR111	GGCCAAGGUUGAGCUCUAGU
5’ Sox2 PP	UGGCUUCCGUGUCAUCGGCU
3’ Sox2 PP	GGUCUAACUUCUCGUCUGUA

Table S4. TaqMan probes for quantitative RT-PCR analysis

Gene	Species	Catalog number
Ubtf	Mouse	Mm00456972_m1
Tfap2c	Mouse	Mm00493473_m1
Sox2	Mouse	Mm00488369_m1
Cdx2	Mouse	Mm01212280_m1

Table S5. SYBR green primer sets for quantitative RT-PCR analysis

Gene	Forward (5’-3’)	Reverse (5’-3’)
hmGfp	CCCAAGGACATCCCTGACTA	GTACACGAAGCAGTCGTCCA
Sox2	GCTCGCAGACCTACATGAACGG	GCCTCGGACTTGACCACAGAGC
